# Efficient Differentiation of Mouse Induced Pluripotent Stem Cells into Alveolar Epithelium Type II with a BRD4 Inhibitor

**DOI:** 10.1155/2019/1271682

**Published:** 2019-12-27

**Authors:** Toru Momozane, Eriko Fukui, Soichiro Funaki, Makoto Fujii, Yuhei Kinehara, Emiko Ito, Shigeru Miyagawa, Yuko Ohno, Yoshiki Sawa, Meinoshin Okumura, Yasushi Shintani

**Affiliations:** ^1^Department of General Thoracic Surgery, Osaka University Graduate School of Medicine, Osaka, Japan; ^2^Department of Mathematical Health Science, Osaka University Graduate School of Medicine, Osaka, Japan; ^3^Department of Respiratory Medicine and Clinical Immunology, Osaka University Graduate School of Medicine, Osaka, Japan; ^4^Department of Cardiovascular Surgery, Osaka University Graduate School of Medicine, Osaka, Japan; ^5^Department of General Thoracic Surgery, National Hospital Organization Toneyama Hospital, Osaka, Japan

## Abstract

Regenerative medicine has continued to progress for lung biology and lung diseases. Efforts have focused on a variety of different applications for pluripotent stem cells. Several groups have reported successful methods for inducing differentiation of induced pluripotent stem cells (iPSCs) into the airway epithelium such as alveolar epithelium type II (ATII). However, differentiation efficiency varies among reports and improvements are needed. In the present paper, we propose a novel method for elimination of residual undifferentiated murine iPSCs using JQ1, a potent inhibitor of bromodomain (BRD) and extraterminal domain (BET) family proteins, for efficient differentiation into ATII. First, the murine iPSC line 20D-17 was induced to differentiate into ATII over a period of 26 days (days 0-26) using previously reported embryoid body seeding and stepwise differentiation methods. mRNA expressions of differentiation markers including surfactant protein C (*Sftpc*) were confirmed by real-time reverse transcription-polymerase chain reaction (RT-PCR) results, and 17% of the cells were shown positive for prosurfactant protein C (proSPC) in flow cytometry analysis. Next, those cells were cultured three-dimensionally in Matrigel for an additional 14 days (days 26-40), during which JQ1 was added for 4 days (days 28-32) to remove residual undifferentiated iPSCs. As a result, on day 40, the mRNA expression level of *Sftpc* in the three-dimensional culture was maintained at the same level as on day 26 and shown to be further increased by the addition of JQ1, with 39% of the cells found to express proSPC, showing that differentiation efficiency could be further increased. Three-dimensional culture with BRD4 inhibition by JQ1 improved the differentiation induction efficiency to ATII by removing residual undifferentiated murine iPSCs during the differentiation induction process.

## 1. Introduction

The lung has a complex structure with major differences in the composition of the epithelium. Several cell types, such as basal cells, club cells, bronchioalveolar stem cells, alveolar epithelium type II (ATII), and distal lung progenitor cells, have also been identified and characterized as endogenous stem and progenitor cells of the epithelium. These cells play a key role in the regeneration of damaged tissues [1–3]. The most distal region of the alveoli includes alveolar epithelium type I (ATI) and ATII. ATI make up the majority of the alveoli and are essential for gas exchange. ATII secrete surfactants and are critical for the maintenance of alveoli. ATII act as endogenous stem and progenitor cells in the alveoli, contributing to alveolar repair by proliferation of ATII and subsequent differentiation into ATI following pulmonary injury [1, 2].

Recently, regenerative medicine has continued to progress for lung biology and lung diseases. The term “stem cells” in lung biology refers to endogenous progenitor cells, pluripotent stem cells, mesenchymal stromal cells, and endothelial progenitor cells as cell therapy agents [4]. Efforts have focused on a variety of different applications for pluripotent stem cells such as embryonic stem cells (ESCs) or induced pluripotent stem cells (iPSCs). These applications include disease modeling, drug discovery, tissue regeneration, and stem cell-based therapies [5]. Although stem cell-based therapy using differentiated cells from iPSCs is challenging [6], such therapy is considered the ultimate goal [7].

Several groups have reported successful methods for inducing differentiation of human and murine iPSCs into airway epithelium cells, including both proximal and distal epithelial cells, using a variety of protocols [8–13]. For inducing differentiation of ATII, most of those studies used embryoid body (EB) seeding or stepwise differentiation methods [14–16], with additional attempts implemented to improve differentiation efficiency. *In vitro* differentiation methods for pluripotent stem cells include a monolayer culture on defined matrices (dissociate seeding method), coculture with heterotypic cell types, and an EB seeding method. The EB seeding method is highly reliable and commonly used [17, 18] since it was first reported as a technique for *in vitro* differentiation induction of mouse embryonal carcinoma cells (ECCs) [19]. The stepwise differentiation method for inducing differentiation into ATII uses the processes of EB, definitive endoderm (DE), anterior foregut endoderm (AFE), and ventralized AFE (VAFE) formation and then differentiation into ATII [14]. However, the efficiency of differentiation into ATII varies, with possible causes including variable maintenance of the differentiation state of the cells after induction and the presence of residual undifferentiated cells following differentiation of iPSCs. Gene expression and protein synthesis change due to various influences, such as flattening of cells in a conventional two-dimensional culture [20, 21]. Three-dimensional cell culturing has been developed in recent years [22] and shown to better maintain the morphology and function of differentiated cells [22, 23]. Matrigel, a complex mixture of multiple proteins of extracellular matrix (ECM) and associated molecules, is widely used for this purpose [24]. On the other hand, a certain number of undifferentiated iPSCs remain in iPSC-derived differentiated cell populations. Undifferentiated murine iPSCs make differentiated derivatives tumorigenic, and even a small number of undifferentiated murine cells can result in teratoma formation *in vivo* [25]. Methods for reducing residual undifferentiated cells have been presented [26] and are broadly classified into those for collecting differentiated cells expressing a differentiation marker, such as sorting-based methods, and methods for removing undifferentiated cells.

The bromodomain (BRD) and extraterminal domain (BET) proteins have been targeted for controlling cancerous cell growth [27]. BRD is a protein domain that recognizes acetylated lysine residues of histones and functions to concentrate regulatory proteins and control the chromatin structure and gene expression [28]. BRD2, BRD3, BRD4, and BRDT are BET family proteins with a BRD repeat sequence and a specific terminal sequence [29]. These proteins play important roles in various intracellular processes such as inflammation-related gene expression, cell division, and virus-host interaction [27]. JQ1, a selective small-molecule inhibitor of BRD4, is a low molecular weight compound that is permeable to the cell membrane and competitively inhibits the binding of the BRD protein (particularly BRD4) to the acetylated lysine residues of histones [30]. JQ1 inhibits cell proliferation of cancer cell-derived cell lines in vitro and in vivo, mainly by downregulating the expression of *cMYC* [31, 32]. In ESCs and mesenchymal stem cells, expression of many pluripotent genes is decreased by BET inhibition using JQ1 [33, 34]. Thus, JQ1 is expected to be applicable for the elimination of residual undifferentiated iPSCs [26].

In the present paper, we propose a novel method to eliminate undifferentiated murine iPSCs with BET inhibition for efficient differentiation into ATII. We investigated whether undifferentiated murine iPSCs were selectively eliminated using medium supplemented with JQ1 after the ATII differentiation protocol.

## 2. Materials and Methods

### 2.1. Cell Lines and Cultures of Murine iPSCs and Murine Lung Epithelial Type II Cells

A murine iPSC line, iPS-MEF-Ng-20D-17 (20D-17) [35], was provided by Riken BRC through the National BioResource Project of MEXT/AMED, Japan. Murine iPSCs were maintained in an undifferentiated state on 0.1% gelatin-coated tissue culture dishes in the absence of serum and feeder cells using ESGRO Complete PLUS Clonal Grade Medium (Millipore, Billerica, MA). Cells were passaged with Accutase (Millipore) and replated every 3-4 days. A murine lung epithelial type II cell line, MLE12, was purchased from ATCC (Manassas, VA) and cultured in HITES (hydrocortisone, insulin, transferrin, estrogen, and selenium) medium (RPMI 1640, 2% FBS, insulin (5 *μ*g/mL), transferrin (10 *μ*g/mL), sodium selenite (30 nM), hydrocortisone (10 nM), *β*-estradiol (10 nM), and HEPES (10 nM)). Cells were passaged with Accutase (Millipore) and replated every 3-4 days.

### 2.2. Murine iPSC Differentiation into ATII *In Vitro*

**Figure 1:**
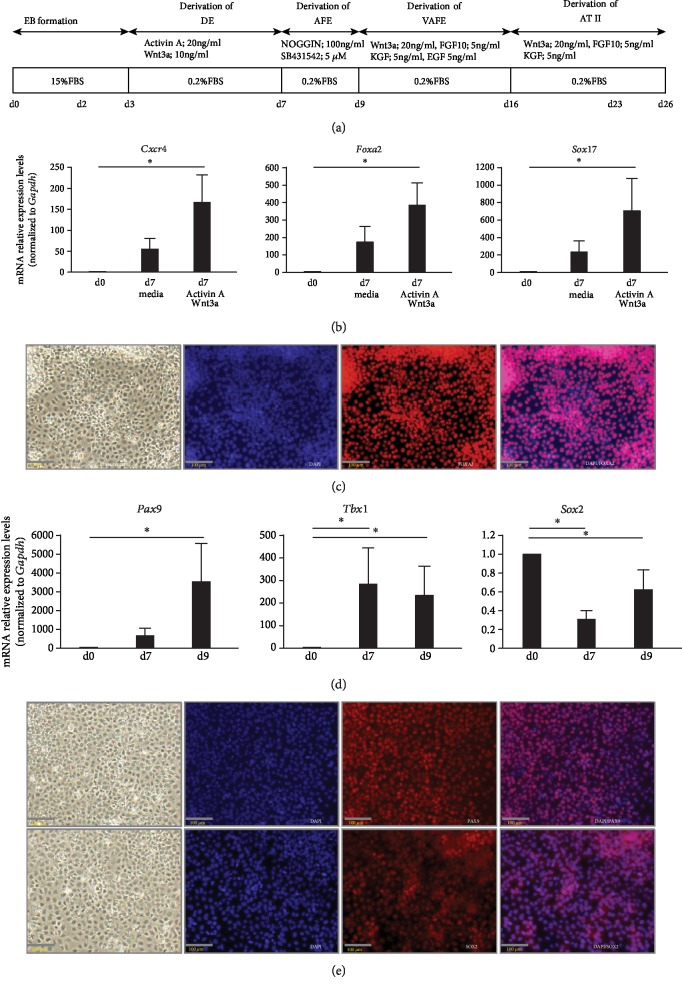
(a) Differentiation protocols for the derivation of ATII using an embryoid body seeding method and a stepwise differentiation method. (b) Quantitative polymerase chain reaction analysis of DE (day 7) (*n* = 5). Expression of *Cxcr4*, *Foxa2*, and *Sox17* mRNA. Expression ratios were normalized to the level of *Gapdh* expression. Expression levels were compared with those of undifferentiated iPSCs (day 0). ^∗^*P* < 0.05. (c) Phase-contrast image and immunofluorescence images of FOXA2 in DE (day 7). Scale bars: 100 *μ*m. (d) Quantitative polymerase chain reaction analysis of AFE (day 9) (*n* = 4). Expression of *Pax9*, *Tbx1*, and *Sox2* mRNA. Expression ratios were normalized to the level of *Gapdh* expression. Expression levels were compared with those of undifferentiated iPSCs (day 0). ^∗^*P* < 0.05. (e) Phase-contrast image and immunofluorescence images of PAX9 and SOX2 in AFE (day 9). Scale bars: 100 *μ*m.

Murine iPSCs were induced to differentiate into ATII as previously described [14–16], with modifications. One thousand iPSCs were resuspended in 100 *μ*L aliquots of differentiation medium (DM) (D-MEM (high glucose); Wako, Osaka, Japan) supplemented with 100 mmol/L nonessential amino acids (Gibco, Grand Island, NY), 2 mmol/L l-glutamine (Gibco), 0.1 mmol/L 2-mercaptoethanol (Gibco), 50 U/mL penicillin, and 50 *μ*g/mL streptomycin (Wako) containing 15% fetal bovine serum (FBS; Sigma-Aldrich, St. Louis, MO) and cultured in 96-well Corning Costar ultra-low attachment multiwell plates (Sigma-Aldrich) for 2 days. Next, individual embryoid bodies (EBs) were plated on 0.1% gelatin-coated tissue culture dishes in DM containing 15% FBS for 1 day. For differentiation into definitive endoderm (DE), the medium was changed to DM containing 0.2% FBS supplemented with 20 ng/mL Activin A (R&D Systems, Minneapolis, MN) and 10 ng/mL Wnt3a (R&D Systems) and then incubated for 4 days with daily medium changes. For anterior foregut endoderm (AFE), the medium was changed to DM containing 0.2% FBS, 100 ng/mL Noggin (R&D Systems), and 5 *μ*M SB431542 (Tocris Bioscience, Bristol, UK) and then incubated for 2 days with daily medium changes. For ventralized AFE (VAFE), the medium was changed to DM containing 0.2% FBS, 20 ng/mL Wnt3a, 5 ng/mL fibroblast growth factor- (FGF-) 10 (R&D Systems), 5 ng/mL keratinocyte growth factor (KGF) (R&D Systems), and 5 ng/mL epidermal growth factor (EGF) (R&D Systems) and then incubated for 7 days with daily medium changes. For ATII, the medium was changed to DM containing 0.2% FBS, 20 ng/mL Wnt3a, 5 ng/mL FGF-10, and 5 ng/mL KGF and then incubated for 10 days with daily medium changes (Figure 1(a)).

### 2.3. RNA Extraction and Real-Time Reverse Transcription Polymerase Chain Reaction

The timing of harvesting cells for RNA extraction and specific markers at each differentiation stage are shown in Supplemental [Supplementary-material supplementary-material-1]. Total RNA was isolated using an RNeasy Kit (Qiagen, Hilden, Germany), and First-Strand cDNA was synthesized from 500-1000 ng total RNA using the PrimeScript RT Master Mix (Takara, Shiga, Japan) according to the manufacturer's manual. Quantitative real-time polymerase chain reaction (qPCR) was performed in duplicate for each sample using the THUNDERBIRD Probe qPCR Mix (Toyobo, Tokyo, Japan) and TaqMan Gene Expression Assay (Applied Biosystems, Foster City, CA) with the CFX96 Real-Time System (Bio-Rad, Hercules, CA). All reactions were started with a cycle of 95°C for 1 minute, followed by 40 cycles of 95°C for 15 seconds and 60°C for 1 minute. The TaqMan Gene Expression Assay identifiers of detected genes were Mm01996749_s1 (*Cxcr4*), Mm01976556_s1 (*Foxa2*), Mm00488363_m1 (*Sox17*), Mm03053810_s1 (*Sox2*), Mm00440629_m1 (*Pax9*), Mm00448949_m1 (*Tbx1*), Mm00447558_m1 (*Nkx2-1*), Mm00485928_m1 (*Atxn1*), Mm00488144_m1 (*Sftpc*), Mm00455678_m1 (*Sftpb*), Mm03053917_m1 (*Klf4*), Mm00487804_m1 (*Myc*), Mm03053917_g1 (*Pou5f1*), and Mm99999915_g1 (*Gapdh*). The level of expression of each gene was normalized to that of *Gapdh*.

### 2.4. Immunofluorescence Analysis

The cells were fixed in 3.7% formalin/phosphate-buffered saline (PBS) for 15 minutes and permeabilized for 15 minutes in 0.2% Triton X-100/PBS. Next, the cells were blocked with 10% normal goat serum blocking solution (Vector Laboratories, Burlingame, CA; S-1000)/PBS for 15 minutes and then stained with a primary antibody at room temperature for 60 minutes. Next, the cells were stained with a secondary antibody at room temperature for 60 minutes and then mounted with VECTASHIELD Hardset Antifade Mounting Medium with DAPI (Vector Laboratories, H-1500). The cells were stained with the following primary antibodies: rabbit anti-forkhead box protein A2 (FOXA2) antibody (1 : 300; Abcam, Cambridge, UK; ab108422), rabbit anti-SRY-box 2 (SOX2) antibody (1 : 100; Abcam; ab97959), rat anti-paired box 9 (PAX9) antibody (1 : 100; Santa Cruz Biotechnology, Santa Cruz, CA; sc-56823), rabbit anti-transcription termination factor 1 (TTF1) antibody (1 : 200; Abcam; ab76013), rabbit anti-prosurfactant protein C (pro-SPC) antibody (1 : 100; Abcam; ab170699), and rabbit anti-pro/mature surfactant protein B (pro-SPB/SPB) antibody (1 : 100; Abcam; ab40876). Stained cells were visualized with the following secondary antibodies: Alexa Fluor 568 goat anti-rabbit IgG (1 : 300; Invitrogen, Carlsbad, CA; A11036) and Alexa Fluor 568 goat anti-rat IgG (1 : 300; Life Technologies, Carlsbad, CA; A11077). Fluorescent photographs were acquired using the confocal laser scanning microscope FSX100 (Olympus, Tokyo, Japan).

### 2.5. Flow Cytometry Analysis of Differentiated VAFE and ATII

The cells were dissociated with Accutase for 15 minutes. The detached cells were diluted in 1% bovine serum albumin/PBS and centrifuged at 900 rpm for 4 minutes. The cell pellets were resuspended in PBS and blocked with purified rat anti-mouse CD16/CD32 (Mouse BD Fc Block) (1 : 50; BD Biosciences, San Jose, CA; 5543142) for 10 minutes on ice. The cells were permeabilized and fixed with Cytofix/Cytoperm fixation and permeabilization solution (BD Biosciences; 51-2090KZ) for 20 minutes, incubated with the primary antibodies for 30 minutes, and, if necessary, incubated with the secondary antibodies for 30 minutes on ice. These cells were analyzed on a FACSAria II flow cytometer (BD Biosciences). To quantify the TTF1-positive cells, the cells were stained with a mouse anti-TTF1 antibody conjugated to APC (1 : 50; Abcore, Ramona, CA; AC12-0362-03). An anti-mouse IgG1 isotype control antibody (APC) (1 : 50; Abcore; 1A-632) was used as an isotype control. For pro-SPC, the cells were stained with a rabbit anti-pro-SPC antibody (2 : 50; Abcam; ab170699), goat anti-rabbit IgG (APC) (1 : 50; Abcam; ab130805), and an anti-rabbit IgG isotype control antibody (APC) (1 : 50, Abcam; ab130805).

### 2.6. Enzyme-Linked Immunosorbent Assay of SPC in Cell Culture Supernatants

Cell culture supernatants were collected; then, a sandwich enzyme-linked immunosorbent assay (Cloud-Clone Corp., Katy, TX) was used for measurement of SPC, according to the manufacturer's protocol. Samples and standards were assayed in duplicate using a CFX96 Real-Time System (Bio-Rad).

### 2.7. Cytostatic Effect of JQ1 on Undifferentiated iPSCs and MLE12

Cell viability was assessed with a premix WST-1 cell proliferation assay system (Takara, Tokyo, Japan). Undifferentiated 20D-17 cells were seeded at a density of 2.0 × 10^3^ cells/well in 96-well plates. The medium was changed every 2 days, and JQ1 (Sigma-Aldrich) was added on the 2nd and 4th days after seeding at concentrations of 0, 0.05, 0.1, 0.2, 0.5, 1, and 2 *μ*M. Measurement was performed on the 6th day, and live cells were evaluated quantitatively. The premix WST-1 reagents were added and incubated with the cells for 1 hour. The absorbance of each well was determined based on formazan product optical densities (450 nm with reference absorbance at 650 nm) using a microplate reader (iMARK, Bio-Rad). A WST assay was also used for MLE12 cells instead of differentiated cells at the minimum inhibitory concentration of JQ1 with undifferentiated iPSCs. MLE12 cells were seeded at a density of 2.0 × 10^3^ cells/well in 96-well plates.

### 2.8. Suppression of Pluripotency of Undifferentiated iPSCs by JQ1

Undifferentiated 20D-17 cells were seeded at a density of 5.0 × 10^3^ cells/well in 12-well plates. Two days after seeding, 0.2 *μ*M JQ1 was added. Total RNA was isolated the next day and the following day, and changes in *Klf4*, *Pou5f1*, *Sox2*, and *cMyc* were measured by qPCR.

### 2.9. SDS-PAGE and Western Blot Analysis

Undifferentiated 20D-17 cells were seeded at a density of 1.0 × 10^5^ cells in a 10 cm dish. Two days after seeding, 0.2 *μ*M JQ1 was added for 24 hours. Cultured cells were treated according to the conditions indicated above; then, proteins were extracted using radioimmunoprecipitation assay buffer (#9806, Cell Signaling Technology, Inc., Danvers, MA). Total proteins were separated by sodium dodecyl sulfate polyacrylamide gel electrophoresis (SDS-PAGE), followed by immunoblotting. Western blotting was performed with the ChemiDoc™ Imaging System (Bio-Rad Laboratories, Inc.). Quantification of protein signals was performed using Image Lab Software, according to the manufacturer's protocol (Bio-Rad Laboratories, Inc.). The primary antibody was anti-Brd4 (dilution 1 : 2000) (Abcam, ab128874). Secondary antibodies were anti-rabbit IgG horseradish peroxidase- (HRP-) conjugated (1 : 2000, Cell Signaling Technology, Inc., 7074). Proteins were visualized using enhanced Western Lightning® Plus-ECL (Perkin Elmer, Inc., Waltham, MA, NEL104001EA), according to the manufacturer's recommendations.

### 2.10. Three-Dimensional Culture of Differentiated ATII with Matrigel and JQ1

On day 26, differentiated cells were dissociated with Accutase, detached single cells were collected, and cells were cultured in three dimensions in Matrigel (Corning, Corning, NY), as described [36], with modifications. In brief, Matrigel at 80 *μ*L/cm^2^ was spread and incubated at 37°C for 30 minutes. Detached cells in SAGM culture medium (Lonza, Verviers, Belgium) containing 2% Matrigel (vol/vol) were seeded on solidified gel. Y27632, an inhibitor of Rho-associated protein kinase, and SB431542 were added as additional factors [11, 37, 38]. Cells were seeded at a density of 2.0 × 10^5^ cells/well in 6-well plates or 1.5 × 10^6^ cells/dish in 10 cm dishes. The SAGM culture medium was changed every 2 days. JQ1 at a concentration of 0.2 *μ*M was also added on days 28 and 30 to remove residual undifferentiated iPSCs. Cultured cells were harvested on day 40. mRNA expression levels were determined on days 26 and 40.

### 2.11. Transmission Electron Microscopy

Differentiated cells were fixed with 2.5% glutaraldehyde in 0.1 M phosphate buffer at pH 7.0 for 1 hour at room temperature, followed by postfixation with 1% osmium tetroxide in 0.1 M phosphate buffer for 1 hour at room temperature. After washing with 0.15 M phosphate buffer, specimens were dehydrated in an ethanol series ranging from 25 to 100% and embedded in Quetol 812 (Nisshin EM, Tokyo, Japan). The samples were cut with a glass knife on an ultramicrotome and poststained with uranyl acetate and lead citrate. Transmission electron microscopic analysis was performed with Hitachi H-7000 (Hitachi High-Technologies Corp., Tokyo, Japan).

### 2.12. Statistical Analysis

All data were obtained from at least three independent experiments. All values are expressed as the mean ± standard deviation. All statistical analyses were conducted using SAS9.4, and the level of significance was 5% (two-tailed). Repeated measurement data were used in this study. However, since cell preservation status and passage number may have influenced the results, we used a linear mixed model. Restricted maximum likelihood was used for estimation. Individual identification and time were included in the model as random effects, and time was included in the model as a fixed effect. The covariance structure assumed a correlation as autoregressive. For comparison between the points of measurement, we used the Dunnett-Hsu method for multiplex adjustment. For comparison at the same point, we used the Tukey-Kramer method for multiplex adjustment.

## 3. Results

### 3.1. Differentiation of Murine iPSCs into ATII

Based on previous reports [14–16], differentiation of murine iPSCs into ATII in vitro was induced with the stepwise differentiation method to generate DE, AFE, VAFE, and subsequently ATII (Figure 1(a)). After EB formation, DE cells were induced by exposure to Activin A and Wnt3a for 4 days. The mRNA expression levels of the DE markers *Cxcr4*, *Foxa2*, and *Sox17* were increased as compared with those of undifferentiated iPSCs on day 7. The addition of Activin A and Wnt3a to DM containing 0.2% FBS (medium) induced stronger differentiation into DE (Figure 1(b)). Microscopically, differentiated cells spread around the EB, and the cells stained positively for FOXA2 (Figure 1(c)). Differentiation of AFE cells from DE cells was induced by exposure to Noggin and SB431542 for 2 days. The mRNA expression levels of the AFE markers *PAX9* and *Tbx1* were increased, while that of *Sox2* showed another increase on day 9 (Figure 1(d)). In comparisons between days 7 and 9, *PAX9* continued to increase, while *Tbx1*, considered to be a specific marker of the thyroid gland rather than the lungs among AFE markers [39], remained nearly unchanged. Cells that differentiated into AFE showed positive staining for PAX9 and SOX2 (Figure 1(e)).

**Figure 2:**
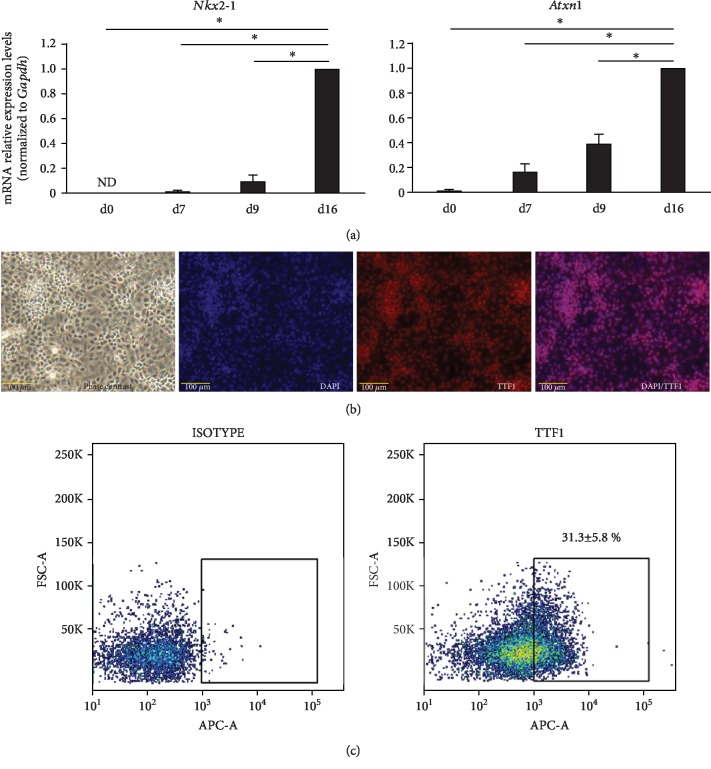
(a) Quantitative polymerase chain reaction analysis of VAFE (day 16) (*n* = 4). Expressions of *Nkx2-1* and *Atxn1* mRNA. Expression ratios were normalized to the level of *Gapdh* expression. Expression levels of *Nkx2-1* and *Atxn1* were compared with those of VAFE (day 16). ^∗^*P* < 0.05. ND: not detected. (b) Phase-contrast image and immunofluorescence images of TTF1 in VAFE (day 16). Scale bars: 100 *μ*m. (c) Flow cytometry analysis of TTF1 expression in VAFE (day 16).

Differentiation of VAFE cells from AFE cells was induced by Wnt3a, FGF-10, KGF, and EGF for 7 days. The mRNA expression levels of both *Nkx2-1* and *Atxn1*, the most representative markers of VAFE, were increased on day 16 (Figure 2(a)). In particular, *Nkx2-1* was not expressed in an undifferentiated state on day 0, while its expression was observed on day 7 and increased over time. Cells that differentiated into VAFE were stained positively for TTF-1 (Figure 2(b)). Additionally, flow cytometry analysis showed that 36.2 ± 5.9% of the total cell population was positive for TTF-1 (Figure 2(c)). Differentiation of ATII from VAFE cells was induced by Wnt3a, FGF-10, and KGF for 10 days.

**Figure 3:**
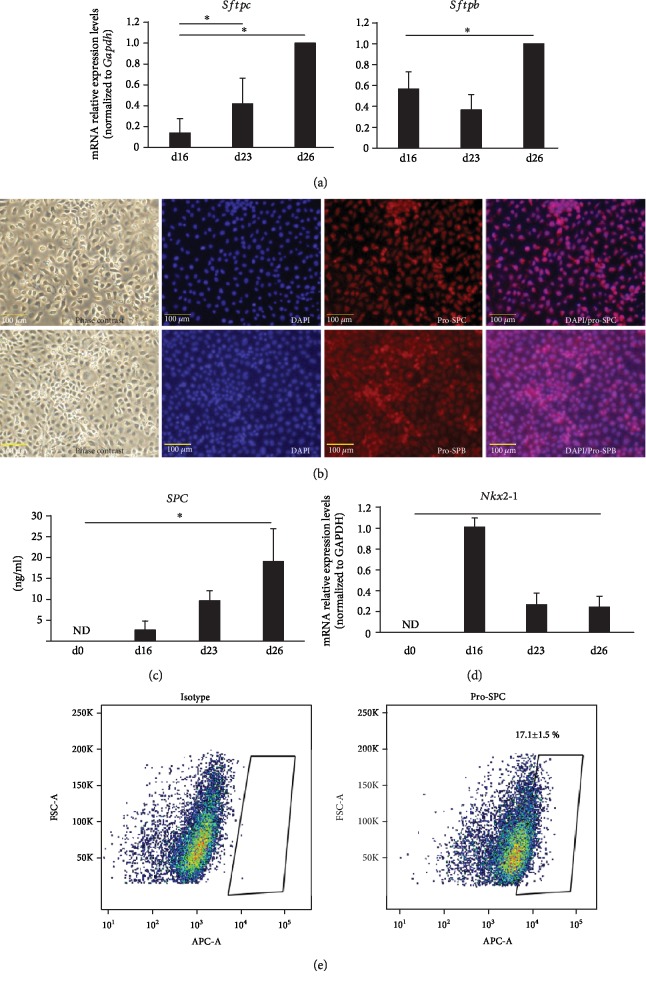
(a) Quantitative polymerase chain reaction analysis of ATII (day 26) (*n* = 5). Expressions of *Sftpc* and *Sftpb* mRNA. Expression ratios were normalized to the level of *Gapdh* expression. Expression levels were compared with those of ATII (day 26). ^∗^*P* < 0.05. (b) Phase-contrast image and immunofluorescence images of pro-SPC and pro-/mature SPB in ATII (day 26). Scale bars: 100 *μ*m. (c) Enzyme-linked immunosorbent assay of SPC in culture supernatant (*n* = 3). ^∗^*P* < 0.05. ND: not detected. (d) Quantitative polymerase chain reaction analysis of ATII (day 26) (*n* = 3). Expressions of *Nkx2-1* mRNA. Expression ratios were normalized to the level of *Gapdh* expression. Expression levels were compared with those of VAFE (day 16). ND: not detected. (e) Flow cytometry analysis of pro-SPC in ATII (day 26).

The mRNA expression levels of both the surfactant protein C (*Sftpc)* and surfactant protein B (*Sftpb)* genes were increased on day 26 (Figure 3(a)). Neither *Sftpc* nor *Sftpb* was expressed until day 9, after which *Sftpc* expression was generally increased over time. On day 23, *Sftpb* expression was temporarily decreased but then showed a strong increase on day 26. Cells that differentiated into ATII were stained positively for pro-SPC and pro-/mature SPB (Figure 3(b)). In addition, the concentration of SPC increased in the cell supernatant (Figure 3(c)). Nkx2-1 expression, a specific marker of VAFE, was decreased but was maintained on day 26 (Figure 3(d)). At this point, various cell groups were mixed in the dish. Flow cytometry analysis showed that 17.1 ± 1.5% of the collected cell population was positive for pro-SPC (Figure 3(e)). These data suggested that undifferentiated murine iPSCs partially differentiated into ATII with the stepwise differentiation method as reported previously [14–16].

### 3.2. Suppression of Proliferation and Pluripotency of Undifferentiated iPSCs by JQ1

**Figure 4:**
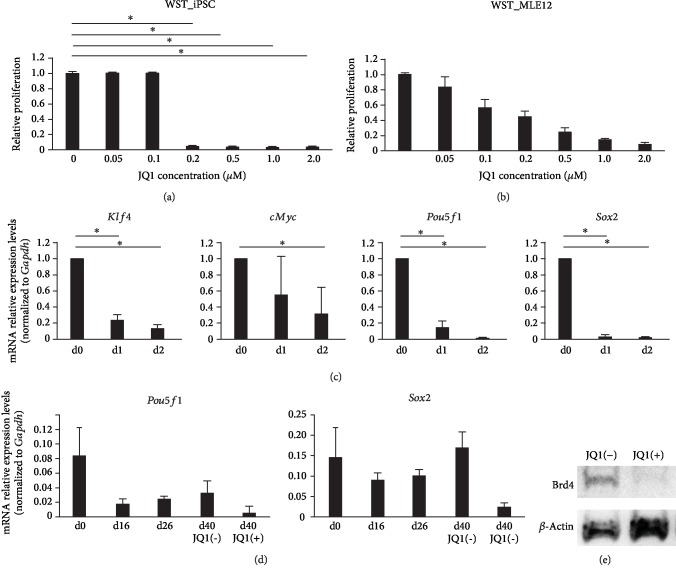
(a) Effect of JQ1 on the WST-1 reduction potential in undifferentiated iPSCs (*n* = 3). Undifferentiated iPSCs were treated with 0, 0.05, 0.1, 0.2, 0.5, 1.0, or 2.0 *μ*M JQ1. ^∗^*P* < 0.05. (b) Effect of JQ1 on WST-1 reduction potential in MLE12 (*n* = 3). Undifferentiated iPSCs were treated with JQ1 at 0, 0.05, 0.1, 0.2, 0.5, 1.0, or 2.0 *μ*M. (c) Quantitative polymerase chain reaction analysis in undifferentiated iPSCs (*n* = 4). Expressions of *Klf4*, *cMyc*, *Pou5f1*, and *Sox2* mRNA. Expression ratios were normalized to the level of *Gapdh* expression. Expression levels were compared with those of undifferentiated cells (day 0). ^∗^*P* < 0.05. (d) Quantitative polymerase chain reaction analysis (*n* = 3). Expressions of *Pou5f1* and *Sox2* mRNA. Expression ratios were normalized to the level of *Gapdh* expression. *Pou5f1* and *Sox2* mRNA expression levels were decreased. (e) Western blot analysis of undifferentiated iPSCs, with the expression of BRD4 protein shown.

We also examined whether undifferentiated murine iPSCs were selectively eliminated using medium supplemented with JQ1 after implementation of the ATII differentiation protocol. First, to confirm the cytostatic effect of JQ1 on undifferentiated iPSCs and MLE12, a WST assay was performed. Cell death of iPSCs was induced with a JQ1 concentration of 0.2 *μ*M or more (Figure 4(a)). Administration of 0.2 *μ*M JQ1 was less cytotoxic towards MLE12 (Figure 4(b)). Next, to investigate the suppression of pluripotency of undifferentiated iPSCs by JQ1, changes in *Klf4*, *Pou5f1*, *Sox2*, and *cMyc* were confirmed. The expression of each was decreased at day 1 after the addition of 0.2 *μ*M JQ1 and then further decreased the following day (Figure 4(c)). Furthermore, to confirm the change in BRD4, JQ1 was administered to undifferentiated iPSCs, which resulted in decreased protein expression of BRD4, as shown by Western blot analysis (Figure 4(e)).

### 3.3. Maintaining ATII and Removal of Residual Undifferentiated iPSCs

**Figure 5:**
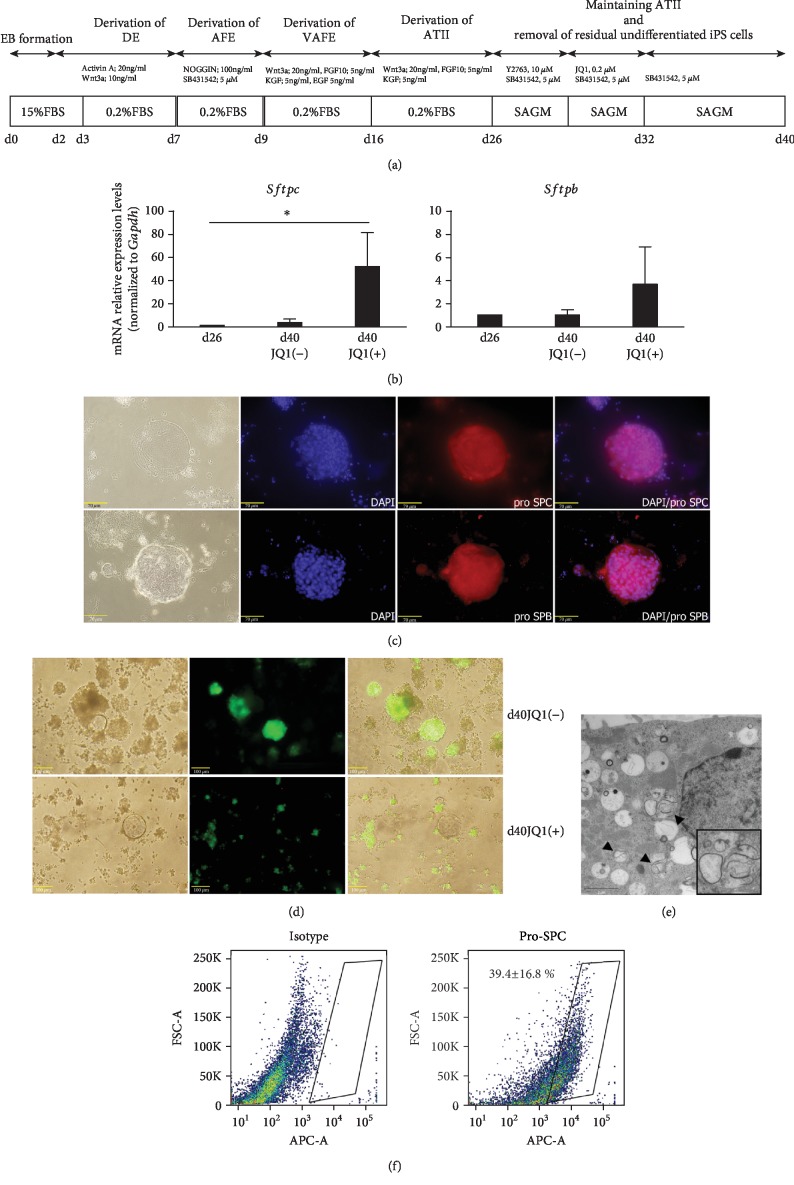
(a) Differentiation protocols for maintaining ATII and removal of residual undifferentiated iPSCs by JQ1. (b) Quantitative polymerase chain reaction analysis (*n* = 4). Expressions of *Sftpc* and *Sftpb* mRNA. Expression ratios were normalized to the level of *Gapdh* expression. Expression levels were compared with those on day 26. ^∗^*P* < 0.05. Three cell groups, day 26, day 40/JQ1(-), and day 40/JQ1(+), were compared. (c) Phase-contrast image and immunofluorescence images of pro-SPC and pro-/mature SPB on day 40. Scale bars: 70 *μ*m. (d) Phase-contrast and fluorescence images of Nanog-GFP on day 40. Nanog-GFP-positive cells were decreased in the JQ1-administered group (day 40/JQ1(+)). Scale bars: 100 *μ*m. (e) Transmission electron microscopy of differentiated ATII on day 40. Lamellar body-like structures were observed (arrowheads). Scale bar: 2 *μ*m. (f) Flow cytometry analysis of pro-SPC on day 40.

On day 26 (Figure 1(a)), differentiated cells were recovered by enzyme treatment with Accutase cell detachment solution and three-dimensional culture with Matrigel was started. JQ1 was also added to remove residual undifferentiated iPSCs (Figure 5(a)). Three cell groups, those obtained on day 26, as well as day 40/JQ1(-) and day 40/JQ1(+) cells, were compared and examined. On day 40, the mRNA expression levels of *Sftpc* and *Sftpb* were shown to be maintained in the three-dimensional culture (day 40/JQ1(-)) at the same level as on day 26, with expression further increased by the addition of JQ1 (day 40/JQ1(+)) (Figure 5(b)). The mRNA expression levels of the pluripotency markers *Klf4*, *Pou5f1*, *Sox2*, and *cMyc* were also measured, and *Pou5f1* and *Sox2* were found to be decreased by JQ1 (Figure 4(d)). Microscopic examinations of cells grown in three-dimensional cultures in Matrigel showed a group of cells that formed spheroids and expressed pro-SPC and pro-/mature SPB (Figure 5(c)), as well as Nanog-GFP-positive cells, thus indicating that residual undifferentiated iPSCs were suppressed by JQ1 (Figure 5(d)). Electron microscopic analysis showed structures in the cytoplasm similar to lamellar-like bodies, which were specific for ATII (Figure 5(e)). Additionally, flow cytometry analysis showed that 39.4 ± 16.8% of the total cell population was positive for pro-SPC (Figure 5(f)). These data suggested that three-dimensional culture with JQ1 enabled effective removal of residual undifferentiated iPSCs.

## 4. Discussion

### 4.1. Differentiation of Murine iPSCs into ATII

The murine iPSC line 20D-17 was shown to differentiate into ATII over a period of 26 days (days 0-26) by the use of EB seeding and stepwise differentiation methods. The presence of ATII was mainly demonstrated by mRNA expression of *Sftpc* and protein expression of pro-SPC. Additionally, mRNA expressions of *Sftpc* and *Sftpb* were also confirmed, and 17% of the cells were positive for pro-SPC on day 26. Several reports have shown that both human and murine iPSCs can be induced to differentiate into the airway epithelium, including proximal and distal epithelial cells [8–13]. Most reports were on induction of differentiation into ATII, and basic differentiation medium and additive trophic factors have been investigated. In particular, the added trophic factors, Activin A and Wnt3a, were used for induction of differentiation into DE. Both the Nodal and canonical Wnt signaling pathways work synergistically to specify the endoderm layer [40]. For induction of differentiation into AFE, NOGGIN and SB431542 were used. Green et al.'s report [14] and that of Longmire et al. [39] demonstrated that differentiation into AFE is induced by dual inhibition of bone morphogenetic protein and transforming growth factor-*β* signaling. In the differentiation of AFE into lung progenitor cells, several molecules and signaling pathways are involved, such as Nodal, transforming growth factor-*β*, bone morphogenetic protein, FGF, retinoic acid, Notch, and Wnt, and better understanding is required [40]. Therefore, various induction methods after AFE have been used. Examination of each cell line may be required. With reference to previous reports [14–16], four trophic factors, Wnt3a, FGF-10, KGF, and EGF, were used to induce differentiation of AFE into VAFE, and three trophic factors, Wnt3a, FGF-10, and KGF, were used to induce ATII from VAFE in this study. Although differences in details such as concentrations of each added factor and the incubation period are present between our study and previous studies, the murine iPSC line (20D-17) could be differentiated into ATII at day 26. Nevertheless, further improvements to increase the differentiation efficiency to ATII are required.

### 4.2. Maintaining a Differentiated State of ATII and Removal of Residual Undifferentiated iPSCs

Cells that differentiated into ATII over a period of 26 days were cultured in three-dimensional cultures in Matrigel for 14 more days (days 26-40), with JQ1 added on 4 of those days (days 28-32), using previously reported methods. As a result, the mRNA expression levels of *Sftpc* and *Sftpb* were maintained in the three-dimensional culture at the same level as seen on day 26, with expression further increased by the addition of JQ1. Furthermore, 39% of the cells expressed pro-SPC.

Three-dimensional cultures can be generally categorized as scaffold-free and scaffold-based culture systems [22, 23]. A scaffold-free culture is synonymous with an aggregate culture, and in stem cell biology, the aggregate is specifically expressed as EB. The EB seeding method, the differentiation induction method used until day 26 in the present study, employs a scaffold-free culture at the beginning. On the other hand, hydrogel technology is a representative scaffold-based culture system and based on various components of the ECM [22, 23]. Some recent studies that induced differentiation of iPSCs into the lung epithelium attempted three-dimensional cultures using Matrigel [8–11]. In the present study, cells had differentiated into ATII by day 26 and then were further cultured three-dimensionally with Matrigel for an additional 14 days and shown to maintain their differentiated state.

Some recent reports have shown high efficiency for differentiation into the airway epithelium by the use of a sorting-based method. Yamamoto et al. demonstrated that carboxypeptidase M (CPM), a surface marker of *Nkx2-1*-positive VAFE cells, had high potential for differentiation to ATII and purified *Nkx2-1*-enriched VAFE cells by the use of CPM sorting and also reported 51% induction efficiency of SFTPC-GFP-positive cells [11]. Hawkins et al. reported CD47 as a positive surface marker and CD26 as a negative surface marker of *Nkx2-1*-positive primordial lung progenitor cells and also purified *Nkx2-1*-positive primordial lung progenitor cells by sorting based on CD47^high^CD26^low^ gating [41]. Sorting-based methods have an important feature of removal of the vast majority of undifferentiated cells from a differentiated culture by sorting the derivatives. To reduce residual undifferentiated cells, several techniques have been suggested, including cell sorting, as described above, and genetic manipulation, such as introduction of suicide genes and interference with tumor progression genes or tumor suppressors. On the other hand, cell transplantation usually requires a large number of differentiated cells, as well as extensive cell purification methods for both laboratory and clinical applications. Therefore, these methods are not considered optimal for removing undifferentiated cells from mixed cell cultures, and attempts have been made to identify small-molecule compounds that selectively inhibit pluripotent stem cells [42, 43]. In the present study, we attempted to remove undifferentiated cells with JQ1. The transcription factor *cMYC* plays essential roles in cell proliferation, apoptosis, and differentiation [44] and is overexpressed in various types of malignant tumors [45, 46]. JQ1 attenuates *cMYC* expression and suppresses cell proliferation, and thus, the effect of JQ1 treatment on Merkel cell carcinoma [47, 48], bladder cancer [49], oral squamous cell carcinoma [50], and hematologic malignancies [44, 51] in which *cMYC* is overexpressed has been reported. Regarding ESCs and mesenchymal stem cells, the expressions of many pluripotent genes, such as *cMyc*, *Klf4*, *Pou5f1*, *Sox2*, *Nanog*, *Lefty*, and *Mof*, have been found to be decreased following BET inhibition with JQ1, though the key target of JQ1 varied in previous reports [33, 34, 52]. As for murine ESCs, Horne et al. reported that JQ1 significantly reduced the expression of *Nanog* but not *cMyc*, and the primary target for JQ1 was found to be *Nanog* [52]. Additionally, Wu et al. reported that BRD4 interact with *Pou5f1* [33]. In our study of murine iPSCs, JQ1 reduced the expression of *Pou5f1* and *Sox2*. BET inhibition reduces the expression of many pluripotency genes, but the target may differ depending on the cell type. Furthermore, Horne et al. reported that morphological differentiation of murine ESC occurred within as few as 6 hours after JQ1 administration, though that change could not be confirmed with the present findings, probably because JQ1 was added to cells subjected to a stepwise method for differentiation to ATII. These results suggest that the response to JQ1 may differ depending on the cell type. During the process of induction of differentiation of iPSCs, elimination of residual undifferentiated iPSCs without harm to differentiated cells is required. Kim et al. reported that BET proteins might be involved in cell cycle regulation of distinct cell types and that their inhibition selectively induced cell death of stem cells, but not other somatic cell types [53]. Also, in our study, JQ1 induced cell death with a relatively low effect on murine lung epithelial type II. Together, these findings suggest that the differentiation state of ATII was maintained by suppressing proliferation of residual undifferentiated cells by JQ1.

Murine iPSCs are expected to be useful for animal experimental models of airway disease modeling and regenerative medicine; thus, generation of pure cultures of murine iPSC-differentiated cells is of great importance. A more robust method for elimination of undifferentiated murine iPSCs from culture is required. Although there is identification of small molecules that perturb vital cells more selectively and efficiently, JQ1 may be an intriguing candidate for efficient and robust elimination of undifferentiated murine iPSCs.

## 5. Conclusions

Three-dimensional culture with the BRD4 inhibitor JQ1 improved the efficiency of induction of differentiation into ATII by removing residual undifferentiated murine iPSCs during the differentiation induction process.

## Data Availability

The research data used to support the findings of this study are included within the article.

## Supplementary Materials

Supplementary Materials Differentiation protocols with timing of cell harvesting for RNA extraction indicated.

## References

[1] Kotton D. N., Morrisey E. E. (2014). Lung regeneration: mechanisms, applications and emerging stem cell populations. *Nature Medicine*.

[2] Hogan B. L. M., Barkauskas C. E., Chapman H. A. (2014). Repair and regeneration of the respiratory system: complexity, plasticity, and mechanisms of lung stem cell function. *Cell Stem Cell*.

[3] Stabler C. T., Morrisey E. E. (2017). Developmental pathways in lung regeneration. *Cell and Tissue Research*.

[4] Wagner D. E., Cardoso W. V., Gilpin S. E. (2016). An official American Thoracic Society workshop report 2015. Stem cells and cell therapies in lung biology and diseases. *Annals of the American Thoracic Society*.

[5] Bouros D., Laurent G. (2012). Regenerative medicine and stem cells: Prometheus revisited. *Respiration*.

[6] Scudellari M. (2016). How IPS cells changed the world. *Nature*.

[7] Lowenthal J., Sugarman J. (2015). Ethics and policy issues for stem cell research and pulmonary medicine. *Chest*.

[8] Chen Y.-W., Huang S. X., de Carvalho A. L. R. T. (2017). A three-dimensional model of human lung development and disease from pluripotent stem cells. *Nature Cell Biology*.

[9] McCauley K. B., Hawkins F., Serra M., Thomas D. C., Jacob A., Kotton D. N. (2017). Efficient derivation of functional human airway epithelium from pluripotent stem cells via temporal regulation of Wnt signaling. *Cell Stem Cell*.

[10] Miller A. J., Hill D. R., Nagy M. S. (2018). *In Vitro* Induction and *In Vivo* Engraftment of Lung Bud Tip Progenitor Cells Derived from Human Pluripotent Stem Cells. *Stem Cell Reports*.

[11] Yamamoto Y., Gotoh S., Korogi Y. (2017). Long-term expansion of alveolar stem cells derived from human IPS cells in organoids. *Nature Methods*.

[12] Wong A. P., Chin S., Xia S., Garner J., Bear C. E., Rossant J. (2015). Efficient generation of functional CFTR-expressing airway epithelial cells from human pluripotent stem cells. *Nature Protocols*.

[13] Ghaedi M., Calle E. A., Mendez J. J. (2013). Human IPS cell-derived alveolar epithelium repopulates lung extracellular matrix. *The Journal of Clinical Investigation*.

[14] Green M. D., Chen A., Nostro M. C. (2011). Generation of anterior foregut endoderm from human embryonic and induced pluripotent stem cells. *Nature Biotechnology*.

[15] Huang S. X. L., Islam M. N., O'Neill J. (2014). Efficient generation of lung and airway epithelial cells from human pluripotent stem cells. *Nature Biotechnology*.

[16] Ghaedi M., Mendez J. J., Bove P. F., Sivarapatna A., Raredon M. S., Niklason L. E. (2014). Alveolar epithelial differentiation of human induced pluripotent stem cells in a rotating bioreactor. *Biomaterials*.

[17] Bratt-Leal A. M., Carpenedo R. L., McDevitt T. C. (2009). Engineering the embryoid body microenvironment to direct embryonic stem cell differentiation. *Biotechnology Progress*.

[18] Pettinato G., Wen X., Zhang N. (2015). Engineering strategies for the formation of embryoid bodies from human pluripotent stem cells. *Stem Cells and Development*.

[19] Martin G. R., Evans M. J. (1975). Differentiation of clonal lines of teratocarcinoma cells: formation of embryoid bodies in vitro. *Proceedings of the National Academy of Sciences of the United States of America*.

[20] Thomas C. H., Collier J. H., Sfeir C. S., Healy K. E. (2002). Engineering gene expression and protein synthesis by modulation of nuclear shape. *Proceedings of the National Academy of Sciences of the United States of America*.

[21] Vergani L., Grattarola M., Nicolini C. (2004). Modifications of chromatin structure and gene expression following induced alterations of cellular shape. *The International Journal of Biochemistry & Cell Biology*.

[22] Knight E., Przyborski S. (2015). Advances in 3D cell culture technologies enabling tissue-like structures to be created in vitro. *Journal of Anatomy*.

[23] Edmondson R., Broglie J. J., Adcock A. F., Yang L. (2014). Three-dimensional cell culture systems and their applications in drug discovery and cell-based biosensors. *ASSAY and Drug Development Technologies*.

[24] Kleinman H. K., Martin G. R. (2005). Matrigel: basement membrane matrix with biological activity. *Seminars in Cancer Biology*.

[25] Liu Z., Tang Y., Lü S. (2013). The tumourigenicity of IPS cells and their differentiated derivates. *Journal of Cellular and Molecular Medicine*.

[26] Masuda S., Miyagawa S., Fukushima S. (2015). Eliminating residual IPS cells for safety in clinical application. *Protein & Cell*.

[27] Taniguchi Y. (2016). The bromodomain and extra-terminal domain (BET) family: functional anatomy of BET paralogous proteins. *International Journal of Molecular Sciences*.

[28] Ntranos A., Casaccia P. (2016). Bromodomains: translating the words of lysine acetylation into myelin injury and repair. *Neuroscience Letters*.

[29] Filippakopoulos P., Picaud S., Mangos M. (2012). Histone recognition and large-scale structural analysis of the human bromodomain family. *Cell*.

[30] Filippakopoulos P., Qi J., Picaud S. (2010). Selective inhibition of BET bromodomains. *Nature*.

[31] Delmore J. E., Issa G. C., Lemieux M. E. (2011). BET bromodomain inhibition as a therapeutic strategy to target C-Myc. *Cell*.

[32] Dawson M. A., Prinjha R. K., Dittmann A. (2011). Inhibition of BET recruitment to chromatin as an effective treatment for MLL-fusion leukaemia. *Nature*.

[33] Wu T., Pinto H. B., Kamikawa Y. F., Donohoe M. E. (2015). The BET family member BRD4 interacts with OCT4 and regulates pluripotency gene expression. *Stem Cell Reports*.

[34] Alghamdi S., Khan I., Beeravolu N. (2016). BET protein inhibitor JQ1 inhibits growth and modulates WNT signaling in mesenchymal stem cells. *Stem Cell Research & Therapy*.

[35] Okita K., Ichisaka T., Yamanaka S. (2007). Generation of germline-competent induced pluripotent stem cells. *Nature*.

[36] Gon H., Fumoto K., Ku Y., Matsumoto S., Kikuchi A. (2013). Wnt5a signaling promotes apical and basolateral polarization of single epithelial cells. *Molecular Biology of the Cell*.

[37] Miyoshi H., Stappenbeck T. S. (2013). In vitro expansion and genetic modification of gastrointestinal stem cells in spheroid culture. *Nature Protocols*.

[38] Bhaskaran M., Kolliputi N., Wang Y., Gou D., Chintagari N. R., Liu L. (2007). Trans-differentiation of alveolar epithelial type II cells to type I cells involves autocrine signaling by transforming growth factor beta 1 through the Smad pathway. *The Journal of Biological Chemistry*.

[39] Longmire T. A., Ikonomou L., Hawkins F. (2012). Efficient derivation of purified lung and thyroid progenitors from embryonic stem cells. *Cell Stem Cell*.

[40] Ghaedi M., Niklason L. E., Williams J. C. (2015). Development of lung epithelium from induced pluripotent stem cells. *Current Transplantation Reports*.

[41] Hawkins F., Kramer P., Jacob A. (2017). Prospective isolation of NKX2-1 – expressing human lung progenitors derived from pluripotent stem cells. *Journal of Clinical Investigation*.

[42] Ben-David U., Gan Q. F., Golan-Lev T. (2013). Selective elimination of human pluripotent stem cells by an oleate synthesis inhibitor discovered in a high-throughput screen. *Cell Stem Cell*.

[43] Kuang Y., Miki K., Parr C. J. C. (2017). Efficient, selective removal of human pluripotent stem cells via ecto-alkaline phosphatase-mediated aggregation of synthetic peptides. *Cell Chemical Biology*.

[44] Kang C., Kim C.-Y., Kim H. S., Park S.-P., Chung H.-M. (2018). The bromodomain inhibitor JQ1 enhances the responses to All-transRetinoic acid in HL-60 and MV4-11 leukemia cells. *International Journal of Stem Cells*.

[45] Delgado M. D., León J. (2010). Myc roles in hematopoiesis and leukemia. *Genes & Cancer*.

[46] Lüscher B., Vervoorts J. (2012). Regulation of gene transcription by the oncoprotein MYC. *Gene*.

[47] Shao Q., Kannan A., Lin Z., Stack B. C., Suen J. Y., Gao L. (2014). BET protein inhibitor JQ1 attenuates Myc-Amplified MCC Tumor growth *in vivo*.

[48] Sengupta D., Kannan A., Kern M. (2015). Disruption of BRD4 at H3K27Ac-enriched enhancer region correlates with decreased c-Myc expression in Merkel cell carcinoma. *Epigenetics*.

[49] Wu X., Liu D., Tao D. (2016). BRD4 regulates EZH2 transcription through upregulation of C-MYC and represents a novel therapeutic target in bladder cancer. *Molecular Cancer Therapeutics*.

[50] Wang L., Wu X., Huang P. (2016). JQ1, a small molecule inhibitor of BRD4, suppresses cell growth and invasion in oral squamous cell carcinoma. *Oncology Reports*.

[51] Ravi D., Beheshti A., Abermil N. (2016). Proteasomal inhibition by Ixazomib induces CHK1 and MYC-dependent cell death in T-cell and Hodgkin lymphoma. *Cancer Research*.

[52] Horne G. A., Stewart H. J. S., Dickson J., Knapp S., Ramsahoye B., Chevassut T. (2015). Nanog requires BRD4 to maintain murine embryonic stem cell pluripotency and is suppressed by bromodomain inhibitor JQ1 together with Lefty1. *Stem Cells and Development*.

[53] Im J. H., In Hwang S., Kim J.-W. (2018). Inhibition of BET selectively eliminates undifferentiated pluripotent stem cells. *Scientific Bulletin*.

