# Supplementary figures and images for: Efficient Differentiation of Mouse Induced Pluripotent Stem Cells into Alveolar Epithelium Type II with a BRD4 Inhibitor
Source: Stem Cells Int. 2019 Dec 27;2019:1271682. doi: 10.1155/2019/1271682 (PMC6948319; doi:10.1155/2019/1271682)

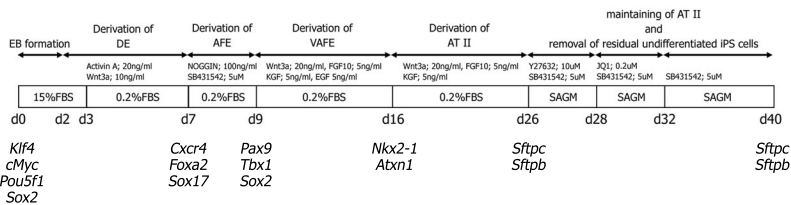

Figure 1B

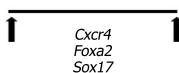

Figure 1D

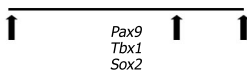

Figure 2A

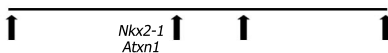

Figure 3A

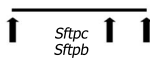

Figure 3D

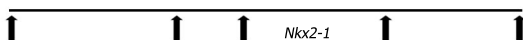

Figure 4D

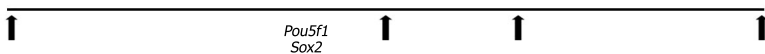

Figure 5B

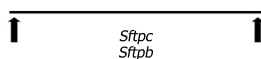

Supplement: Supplementary Materials — Differentiation protocols with timing of cell harvesting for RNA extraction indicated. [file 1271682.f1.pdf]
